# Folic acid Ameliorates the Declining Quality of Sodium Fluoride-Exposed Mouse Oocytes through the *Sirt1*/*Sod2* Pathway

**DOI:** 10.14336/AD.2022.0217

**Published:** 2022-10-01

**Authors:** Xiaoyuan Lin, Beibei Fu, Yan Xiong, Shiyao Xu, Jin Liu, Mohamed Y. Zaky, Dan Qiu, Haibo Wu

**Affiliations:** ^1^School of Life Sciences, Chongqing University, Chongqing 401331, China.; ^2^School of Pharmaceutical Sciences, Chongqing University, Chongqing, China.; ^3^Molecular Physiology Division, Zoology Department, Faculty of Science, Beni-Suef University, Beni-Suef, Egypt.; ^4^Center of Space Exploration, Ministry of Education, Chongqing University, Chongqing, China

**Keywords:** sodium fluoride, folic acid, oocyte, reactive oxygen species, mitochondrial damage, apoptosis

## Abstract

Excessive sodium fluoride (NaF) intake interferes with reproductive function in humans and animals; however, strategies to prevent these effects are still underexplored. Here, we showed that *in vivo* and *in vitro* supplementation of folic acid (FA) efficaciously improved the quality of NaF-exposed oocytes. FA supplementation not only increased ovulation of oocytes from NaF-treated mice but also enhanced oocyte meiotic competency and fertilization ability by restoring the spindle/chromosome structure. Moreover, FA supplementation could exert a beneficial effect on NaF- exposed oocytes by restoring mitochondrial function, eliminating reactive oxygen species accumulation to suppress apoptosis. We also found that FA supplementation restored the defective phenotypes in oocytes through a *Sirt1*/*Sod2*-dependent mechanism. Inhibition of *Sirt1* with EX527 abolished the FA-mediated improvement in NaF-exposed oocyte quality. Collectively, our data indicated that FA supplementation is a feasible approach to protect oocytes from NaF-related deterioration.

As a highly active environmental element, fluorine exists widely in environment as organic or inorganic compounds. In recent years, the potential relationship between long-term NaF exposure and reproductive toxicity has attracted much attention in human and animals [1-5]. The effects of excessive NaF ingestion include reduced pregnancy rate and fertility, damage to ovaries and uterine structures [6], and blocking of meiotic progression as well as disturbed spindle assembly in oocytes [7]. A previous study showed that NaF exposure induced oxidative stress through depletion in levels of various antioxidants, including superoxide dismutase (Sod) [8]. NaF has also been reported to induce apoptosis through a caspase-mediated pathway [9-12]. However, strategies to sustain oocyte quality with NaF exposure have yet to be fully explored.

FA is a synthetic form of folate, which is used in many supplements and fortified staple foods. Several studies have demonstrated its role in reducing the risk of fetal neural tube defects [13], and the World Health Organization recommends the supplementation with FA until 12 weeks of gestation [14]. In women undergoing *in vitro* fertilization (IVF), FA supplements increase the concentration of folate in follicular fluid, thereby decreasing homocysteine concentration and suppressing its detrimental effects on oocyte quality [15]. Research conducted over recent decades has indicated that FA is be beneficial for oocyte maturation and quality [16-19]. Supplementation of FA during oocyte growth may also influence embryo epigenetic reprograming and alter the levels of important imprinted genes [20]. However, despite the great progress seen in FA application, its effects on NaF-induced reproductive damage in females have yet to be fully determined.

In the present study, we showed that both *in vivo* and *in vitro* FA supplementation rescued the quality of NaF-exposed oocytes, enhancing the maturation, fertilization ability, and subsequent developmental potential. We further showed that FA supplementation restored NaF-exposed oocyte quality by recovering mitochondrial function, which in turn, decreased reactive oxygen species (ROS) accumulation and suppressed oocyte apoptosis. Moreover, we found that this protective effect was mediated by a *Sirt1*/*Sod2*-dependent mechanism.

## MATERIALS AND METHODS

### Ethics statement

This study was carried out in strict accordance with the guidelines for the care and use of animals of Chongqing University. All animal experiment procedures were approved by the Animal Ethics Committees of the School of Life Sciences, Chongqing University.

### Mice

Six- to 8-week-old female C57BL/6 mice were purchased from Tengxin Biotechnology Company (Chongqing, China). The mice were kept under controlled conditions, with a temperature (20-23 °C) and illumination (12 h light-dark cycle), and with free access to water and food throughout the study. All animal study protocols were reviewed and approved by Chongqing University School of Life Sciences review boards for animal studies.

### NaF and FA treatment

For the *in vivo* experiment, mice were administered with NaF (Sigma-Aldrich, St. Louis, USA) or NaF+FA (Sigma-Aldrich) for 20 days, and then intraperitoneally injected with 10 mg/kg MTX (MedChemExpress, New Jersey, USA) every other day for 8 days as previously described [21]. For blocking FA receptors with antibodies, anti-FOLR1 (SAB2104804, Sigma-Aldrich) and anti-FOLR2 (SAB1307181) antibodies were diluted in PBS in a ratio of 1:1 and intraperitoneally injected into mice, and the injection was repeated every 3 days until superovulation. After the second injection, the mice were concurrently treated with NaF+FA for 20 days.

For the *in vitro* treatment, NaF (Sigma-Aldrich) was diluted in M2 medium (Sigma-Aldrich) to produce a final concentration of 60 μg/mL. FA was diluted in NaF-containing culture medium to yield a final concentration of 100 μg/mL. NaF and FA were supplemented to the maturation medium at the beginning of the culture, followed by culture to maturation.

### Oocyte collection and culture

For the *in vivo* experiment, female mice were randomly divided into 3 groups (n = 30 in each group): control (treated with ultrapure drinking water), NaF (treated with 60mg/L NaF in drinking water), and NaF+FA (60mg/L NaF +100mg/L FA in drinking water). The concentrations of NaF used in this study were based on the toxicity dose of NaF dissolved in deionized water and the LD50 value of 54.4 mg F/kg body weight has been tested in mice [22, 23]. Mice were injected with pregnant mare serum gonadotropin (PMSG) followed by intraperitoneal injection with 10 IU human chorionic gonadotropin (hCG) after 48 h. Fifteen hours post injection, mice were sacrificed by cervical dislocation, and cumulus-oocyte complexes were collected from ampulla of fallopian tubes. CCs were removed by a brief incubation in 1mg/ml hyaluronidase, and PBE rates were calculated.

For the *in vitro* experiment, female mice were injected with 10 IU PMSG, as described above. Forty-eight hours post injection, full-grown GVs were obtained by puncturing antral ovarian follicles with a needle. GV oocytes were cultured in M16 medium (Sigma-Aldrich) containing NaF, NaF+FA, or NaF+FA+EX527 at 37 °C in 5% CO_2_ for maturation and further analysis.

### IVF and embryo culture

Spermatozoa were isolated from caudae epididymides of 12-week-old male mice and were added to ovulated oocytes in 100 μL of IVF medium (Vitrolife Sweden AB, V. Frölunda, Sweden) covered by paraffin oil for 6 h at 37 °C in 5% CO2. The presence of two pronuclei was considered as successful fertilization. The embryos were cultured in KSOM (Sigma-Aldrich) at 37 °C in humidified air with 5% CO_2_.

### Collection of ovaries, mural granulosa cells (MGCs), and cumulus cells (CCs)

MGCs were obtained from the ovaries of mice using the follicle puncture method with a fine needle. CCs were freed from oocytes mechanically by pipetting oocytes in M2 medium. Ovaries for histological analysis were obtained from each group of mice and fixed in 4% paraformaldehyde overnight at 4 °C, dehydrated, and embedded in paraffin. Ovaries were then sectioned at a thickness of 8 μm for hematoxylin and eosin (H&E) staining.

### Immunofluorescence microscopy

Oocytes were fixed in 4% paraformaldehyde in phosphate-buffered saline (PBS) for at least 30 min and permeabilized in Enhanced Immunostaining Permeabilization Buffer (P0097, Beyotime, Shanghai, China) for 15 min at RT. Afterwards, oocytes were blocked in QuickBlock™ Blocking Buffer for Immunol Staining (P0260, Beyotime) for 8 h at 4 °C and incubated with anti-tubulin antibody (1:1000, ab6160, Abcam, Cambridge, England) overnight at 4 °C. After washing in PBS, oocytes were incubated with the corresponding secondary antibody at RT for 2 h. The oocytes were then counterstained with DAPI for 10 min and observed under a confocal fluorescence microscope.

For the measurement of fluorescence intensity, signals from both treatment and control oocytes were acquired by performing the same immunostaining procedure. Image J (NIH, Bethesda, MD, USA) was used to define a region of interest, of which the average fluorescence intensity per unit area was determined. The average values of all measurements were used to compare the final average intensities between the treatment and control groups.

### Annexin-V staining

For Annexin-V staining, oocytes were stained with an Annexin V-Fluorescein Isothicyanate (FITC) Apoptosis Detection kit (BD, Biosciences.). After washing in PBS, the viable oocytes were stained for 30 min in the dark with 90 μL of binding buffer supplemented with 10 μL Annexin-V-FITC. The oocytes were then washed three times in PBS containing 0.1% BSA and observed under a fluorescence microscope.

### Mitochondria distribution

MitoTracker Deep Red (Invitrogen, CA, USA) was used to evaluate the distribution of mitochondria in oocytes. Briefly, oocytes were incubated in pre-warmed staining solution at 37 °C for 30 min. After washing for three times, oocytes were counterstained with DAPI to visualize nuclei. The images were observed and captured using a microscope. Mitochondria distribution manner were separated into three typical groups including homogenous, perinuclear, and clustering, according to previous studies [24, 25].

### Measurement of mitochondrial DNA (mtDNA) copy numbers

MtDNA copy number in oocytes was assessed as previously described [26]. A single oocyte was put into a PCR tube with 20 μL lysis buffer (50 mM Tristan, 0.1 mM EDTA, 100 μg/mL Proteinase K and 0.5% Tween-20), followed by incubation at 55 °C for 30 min and 95 °C for 10 min. To obtain purified DNA, PCR products were amplified with a mouse mtDNA-specific primer and ligated into a T-vector.

### Determination of ROS levels, GSH levels, and Cathepsin B activity

An ROS Assay Kit (Beyotime) was used to determine the ROS level in living oocytes. Oocytes were incubated with oxidation-sensitive fluorescent probe [dichloro-fluorescein (DCFH)] for 30 min at 37 °C. The oocytes were washed three times in PBS containing 0.1% BSA and observed under a fluorescence microscope. GSH levels were determined by incubating oocytes in culture medium containing 10 μM 4-chloromethyl-6.8-difluoro-7-hydroxycoumarin (CMF_2_HC, Invitrogen) for 30 min and observed under a fluorescence microscope. Cathepsin B activity was measured using a commercial Magic Red cathepsin B Assay Kit (Immunochemistry Technologies LLC, MN, USA), according to the manufacturer’s protocols. Image J was used to analyze fluorescence intensities of oocytes.

### Mitochondrial membrane potential (MMP)

MMP was determined using a Mitochondrial Membrane Potential Assay Kit with JC-1 (Beyotime). Briefly, oocytes were cultured in 500 μL medium with 500 μL JC-1 for 30 min at 37 °C, followed by washing with buffer for 3 times and observed under a fluorescence microscope.

### Evaluation of total ATP content

Total ATP content in a pool of around 40 oocytes was determined using a Bioluminescent Somatic Cell Kit (Sigma-Aldrich), following the manufacturer’s instructions.

### Real-time quantitative PCR

A total of 50 oocytes from treatment or control groups were collected. Total RNA was isolated using a RNeasy Micro Kit (Qiagen, Germantown, USA) and reverse transcribed using the iScript cDNA Synthesis Kit (BioRad, California, USA). The mRNA levels were quantified with an SsoAdvanced Universal SYBR Green Supermic Kit (BioRad) according to the manufacturer’s instructions. Data were analyzed using the threshold cycle (2^-ΔΔ^*^CT^*) method. All primer sequences used for qRT-PCR were listed in Supplementary Table 1.

**Figure 1.**
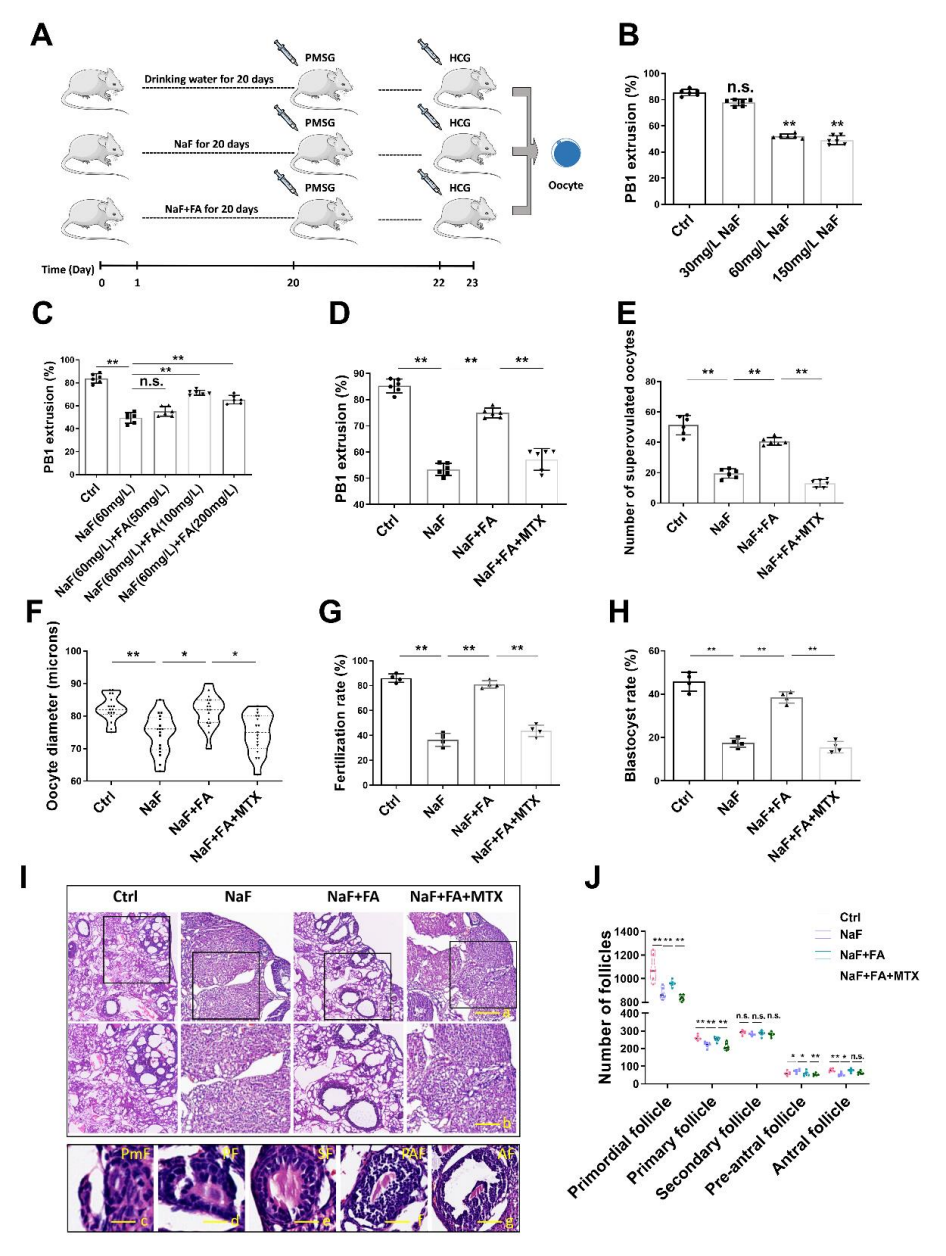
**Effect of FA supplementation on meiotic maturation and quality of oocytes in NaF-treated mice**. (**A**) Timeline diagram of NaF or NaF+FA administration to mice and hormone injection for superovulation of oocytes. (**B**) PBE rates of *in vivo*-matured oocytes obtained from mice administered with water, or NaF at concentrations of 30mg/L, 60mg/L, and 150mg/L (n=6 in each group; each point is cumulative data from one independent experiment; one-way ANOVA; Turkey’s multiple comparison test). **, p < 0.01; n.s., not significant. (**C**) PBE rates of oocytes from mice administered with water, NaF (60mg/L), or NaF (60mg/L) +FA at concentrations of 50mg/L, 100mg/L, and 200mg/L (n=6 in each group; each point is cumulative data from one independent experiment; one-way ANOVA; Turkey’s multiple comparison test). **, p < 0.01; n.s., not significant. (**D**) PBE rates of oocytes from mice administered with water, NaF (60mg/L), FA (100mg/L), or MTX (10 mg/kg) (n=6 in each group; each point is cumulative data from one independent experiment; one-way ANOVA; Turkey’s multiple comparison test). **, p < 0.01. (**E**) Ovulated oocytes were counted in Ctrl, NaF, FA, and MTX mice (n=6 in each group; each point represents one mouse; one-way ANOVA; Turkey’s multiple comparison test). **, p < 0.01. (**F**) Diameter of oocytes from mice in Ctrl, NaF, FA, and MTX groups (n=17 in each group; one-way ANOVA; Turkey’s multiple comparison test). *, p < 0.05; **, p < 0.01. (**G**) *In vitro* fertilization rates of oocytes in Ctrl, NaF, FA, and MTX groups (n=4 in each group; each point is cumulative data from one independent experiment; Bootstrap method). **, p < 0.01. (**H**) Blastocyst rates of oocytes in Ctrl, NaF, FA, and MTX groups (n=4 in each group; each point is cumulative data from one independent experiment; Bootstrap method). **, p < 0.01. (**I**) Representative images of ovarian sections from mice treated with NaF, FA, and MTX following staining with H&E. Scale bars: a, 250 μm; b, 100 μm; c: 10μm; d: 20 μm; e: 50 μm; f: 120 μm; g: 150μm. PmF, primordial follicle; PF, primary follicle; SF: secondary follicle; PAF: pre-antral follicle; AF: antral follicle. (**J**) Number of follicles at different developmental stages in ovaries of female mice in Ctrl, NaF, FA, and MTX groups (n=6 in each group; two-way ANOVA; Turkey’s multiple comparison test). *, p < 0.05; **, p < 0.01; n.s., not significant.

### Western Blotting

Oocyte, MGC and CC lysates were prepared for protein sample isolation. The following antibodies were used to detect protein levels: anti-Bcl 2 (15071, Cell Signaling Technology, CST, Danvers, MA, USA), anti-Bax (89477, CST), anti- cleaved caspase 3 (9654, CST), and anti-cleaved caspase 9 (20750), anti-Sirt1 antibody (SAB570004, Sigma-Aldrich) and anti-Sod2 antibody (AB10346, Sigma-Aldrich). Western blot analysis was performed as described previously [27] using the according protocols.

### Flow cytometry

Annexin V staining, paired with propidium iodide (PI), was used to identify apoptotic MGCs or CCs with an Annexin V- FITC Apoptosis Detection Kit. Flow cytometry analysis was performed according to standard procedures.

### Statistical analysis

Datasets were subjected to the D’Agostino & Pearson test (N≥8) or the Shapiro-Wilk test (6≤N<8). If all the groups passed this test, datasets were analyzed with either two-tailed Student’s t test, one-way ANOVA (3 or more groups), or two-way ANOVA (2×2 study design), with between group comparisons made by Turkey’s multiple comparison test, as indicated in the Figure legends. Non-parametric datasets (N<6 or data with non-normal distribution) were analyzed by Bootstrap method.

All statistical analyses were performed using GraphPad Prism version 8.3.0 or R. *, p < 0.05; **, p < 0.01; n.s., not significant.

## RESULTS

### FA supplementation recovers meiotic maturation and the quality of oocytes in NaF-treated mice

First, we investigated whether FA supplementation restored oocyte quality in NaF-treated mice. Mice were administered drinking water, NaF, or NaF+FA for 20 consecutive days and then received hormones on day 20 and 22 for superovulation (Fig. 1A). Meiotic progression was assessed by calculating the first polar body extrusion (PBE) rate. To determine the optimal dose, different concentrations of NaF, namely, 30, 60, and 150 mg/L, were administered; 60 mg/L NaF was chosen for subsequent study (Fig. 1B). Similarly, different concentrations of FA, namely, 50, 100, and 200 mg/L, were administered together with 60 mg/L NaF; 100 mg/L FA was chosen for subsequent study (Fig. 1C). As expected, treatment with NaF+FA rather than with NaF+FA and methotrexate (MTX, a FA inhibitor) [28] increased PBE rate (Fig. 1D). Consistently, the number of superovulated oocytes and the diameter of oocytes in NaF+FA were also significantly increased as compared with those in NaF or NaF+FA+MTX group (Fig. 1E-F). The effect of NaF on fertilization potential and embryo development was also assessed. The fertilization rate and blastocyst rate were remarkably improved in the NaF+FA group as compared with the NaF group (Fig. 1G-H). As expected, treatment of NaF+FA+MTX decrease the fertilization rate and blastocyst rate compared with the NaF+FA group (Fig. 1G-H). In addition, we assessed follicle development by ovary sections. We observed that FA supplementation increased the number of follicles to some extent (Fig. 1I-J), supporting the result that superovulated oocytes from NaF-treated mice grew in number following FA supplementation. Thus, these observations indicated that FA could at least partially restore meiotic maturation and the quality of oocytes in NaF-treated mice.

**Figure 2.**
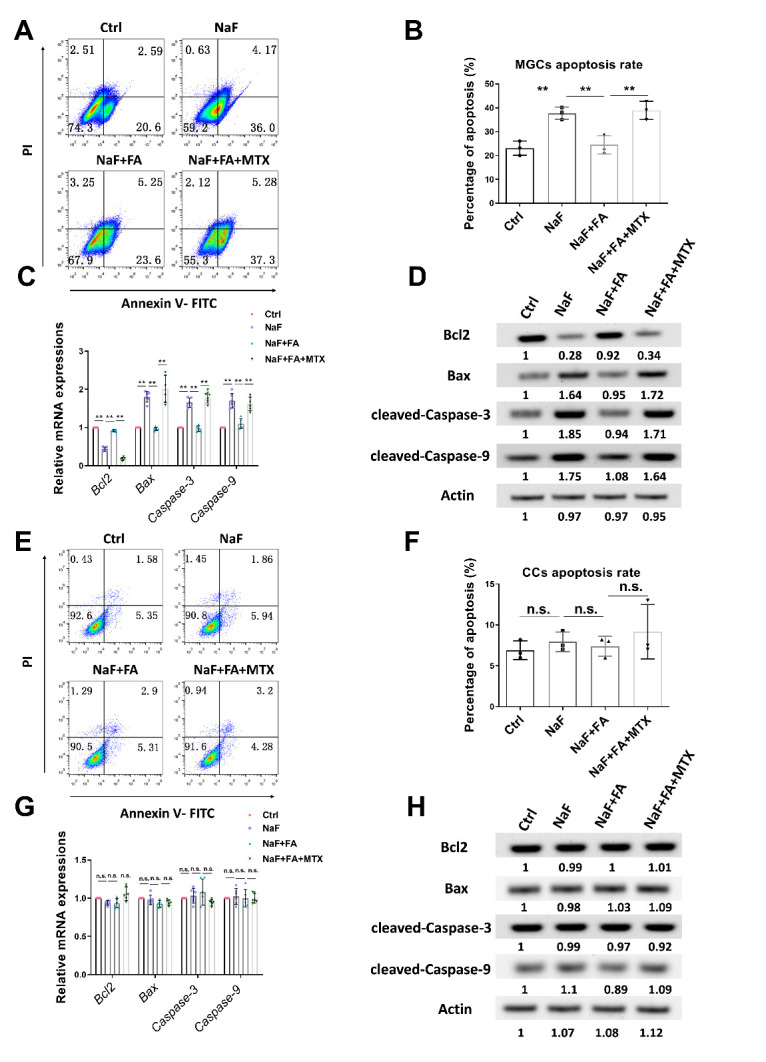
**Effects of FA on apoptosis of mural granulosa cells (MGCs) and cumulus cells (CCs) in NaF-treated mice**. (**A**) Apoptosis of MGCs in Ctrl, NaF, FA, and MTX groups was measured by flow cytometry. (**B**) Apoptosis rate of MGCs from mice exposed to NaF, FA, and MTX (n=3 in each group; each point represents one independent experiment; Bootstrap method). **, p < 0.01. (C-D) qRT-PCR (C; n=6 in each group; each point represents one technical replicate; two-way ANOVA; Turkey’s multiple comparison test; *, p < 0.01) and Western Blotting (D) were used to detect *Bcl2*, *Bax*, *Caspase-3*, and *Caspase-9* expression levels in MGCs. Fifty oocytes for each group were collected for qRT-PCR and 100 oocytes for each group were collected for Western Blotting. (**E**) Apoptosis of CCs in Ctrl, NaF, FA, and MTX groups was measured by flow cytometry. (**F**) Apoptosis rate of CCs from mice exposed to NaF, FA, and MTX (n=3 in each group; each point represents one independent experiment; Bootstrap method). n.s., not significant. (G-H) qRT-PCR (G; n=6 in each group; each point represents one technical replicate; two-way ANOVA; Turkey’s multiple comparison test; n.s., not significant.) and Western Blotting (H) were used to detect *Bcl2*, *Bax*, *Caspase-3*, and *Caspase-9* expression levels in CCs. Fifty oocytes for each group were collected for qRT-PCR and 100 oocytes for each group were collected for Western Blotting.

**Figure 3.**
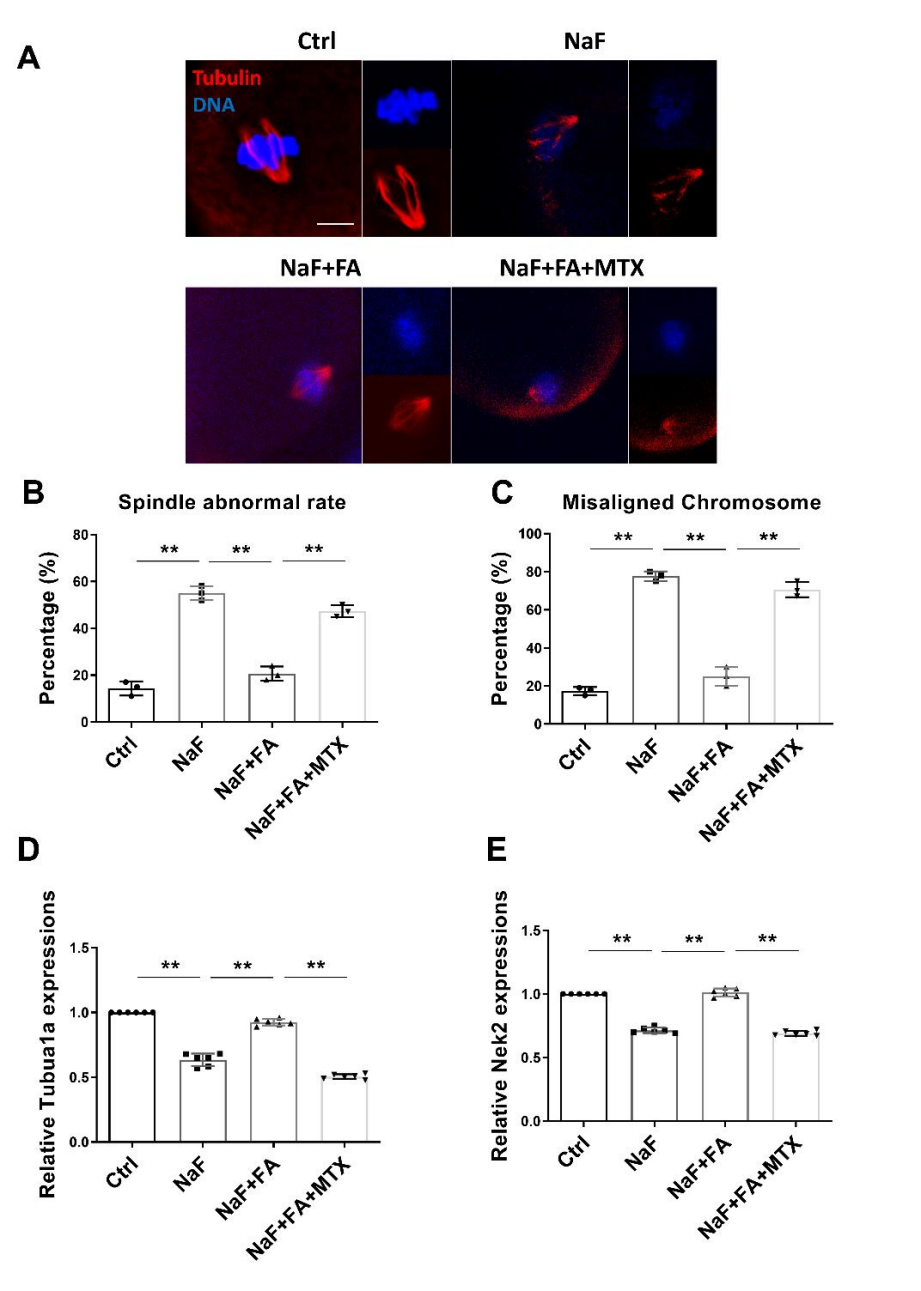
**FA restores spindle disruption and kinetochore-microtubule attachment in NaF-treated mice**. (**A**) Representative images of spindle morphologies and alignment of chromosomes in Ctrl, NaF, FA, and MTX groups. Scale bar, 10μm. Red, α-tubulin; blue, DNA. (**B**) Percentage of aberrant spindles in Ctrl, NaF, FA, and MTX groups (n=3 in each group; each point is cumulative data from one independent experiment; Bootstrap method). **, p < 0.01. (**C**) Percentage of misaligned chromosomes in Ctrl, NaF, FA, and MTX groups (n=3 in each group; each point is cumulative data from one independent experiment; Bootstrap method). **, p < 0.01. (D-E) Expressions of genes implicated in spindle formation/ attachment and meiotic execution (*Tuba1a* and *Nek2*) in oocytes were detected using qRT-PCR (n=6 in each group; each point represents one technical replicate; one-way ANOVA; Turkey’s multiple comparison test; **, p < 0.01). Fifty oocytes for each group were collected for qRT-PCR.

### FA supplementation suppressed oocyte apoptosis in mural granulosa cells (MGCs) and cumulus cells (CCs) in NaF-treated mice

As important components of ovary, MGCs and CCs were collected for flow cytometry and assays of apoptotic markers. In MGCs, FA treatment significantly decreased the proportion of apoptotic cells compared with that detected in the NaF-treated group, which was restored by MTX supplementation (Fig. 2A-B). Similarly, qRT-PCR and Western Blotting results showed that, FA treatment decreased the levels of apoptotic markers, including Bax, Caspase-3, and Caspase-9, while increasing the level of Bcl2 (Fig. 2C-D, Supplementary Fig. 1A-D). However, the results of flow cytometry (Fig. 2E-F) and assays for apoptotic genes (Fig. 2G-H, Supplementary Fig. 1E-H) showed that apoptosis in CCs did not differ between treatments. Collectively, these results suggested that FA inhibited NaF-induced apoptosis in MGCs, whereas NaF treatment did not affect the apoptosis of CCs.

**Figure 4.**
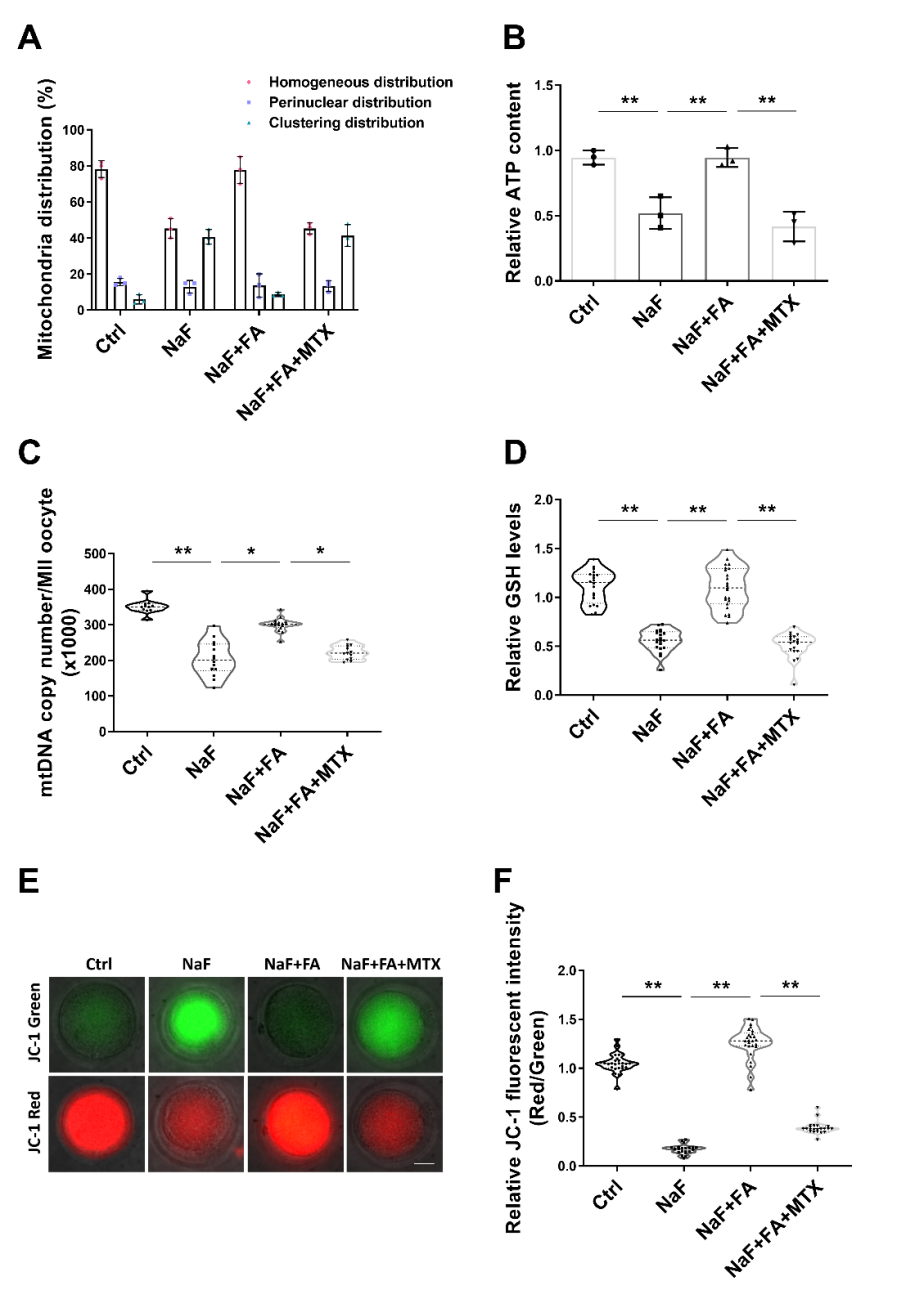
**FA treatment improves mitochondrial function in NaF-treated mice**. (**A**) Rates of homogeneous, perinuclear, and clustering mitochondrial distribution pattern in metaphase II oocytes in Ctrl, NaF, FA, and MTX groups (n=3 in each group; each point is cumulative data from one independent experiment; Bootstrap method). (**B**) Relative ATP content of oocytes in Ctrl, NaF, FA, and MTX groups (n=3 in each group; each point represents one independent experiment; Bootstrap method). **, p < 0.01. (**C**) MtDNA copy number of oocytes in Ctrl, NaF, FA, and MTX groups (n=11, 14, 16, 13; one-way ANOVA; Turkey’s multiple comparison test). *, p < 0.05; **, p < 0.01. (**D**) Relative GSH levels of oocytes in Ctrl, NaF, FA, and MTX groups (n=20, 20, 23, 19; Bootstrap method). **, p < 0.01. (**E**) Mitochondrial membrane potential was calculated as the ratio of red fluorescence, which corresponds to activated mitochondria (J-aggregates), to green fluorescence, which corresponds to less-activated mitochondria (J-monomers). Scale bar, 20μm. (**F**) Relative fluorescence intensity of the mitochondrial membrane potential in Ctrl, NaF, FA, and MTX groups (n=25, 21, 26, 22; Bootstrap method). **, p < 0.01.

**Figure 5.**
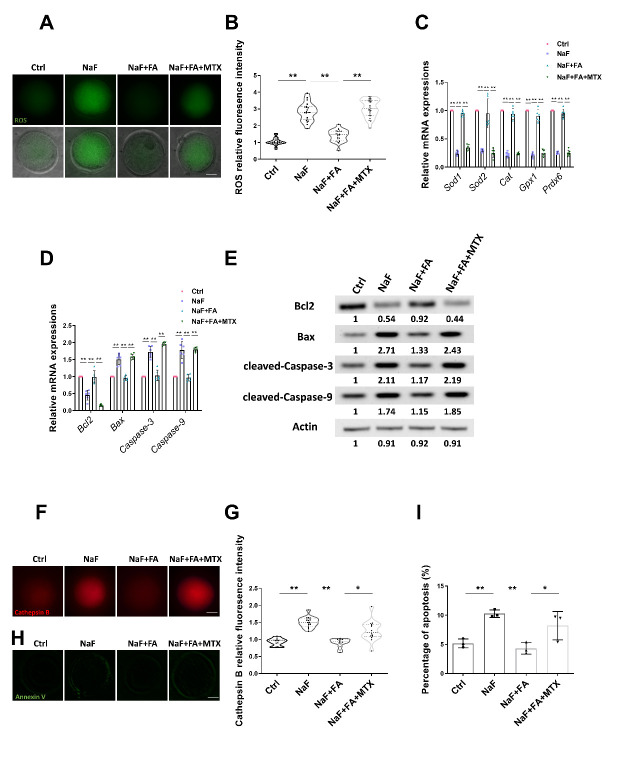
**FA attenuates ROS production to suppress cell apoptosis in NaF-treated mice**. (**A**) ROS levels of oocytes in Ctrl, NaF, FA, and MTX groups. Scale bar, 20μm. (**B**) Relative fluorescence intensity of ROS levels in Ctrl, NaF, FA, and MTX groups (n=24, 21, 21, 23; one-way ANOVA; Turkey’s multiple comparison test). **, p < 0.01. (**C**) qRT-PCR was used to detect oxidative stress-related genes, including *Sod1*, *Sod2*, *Cat*, *Gpx1*, and *Prdx6*. Fifty oocytes for each group were collected for qRT-PCR (n=6 in each group; each point represents one technical replicate; one-way ANOVA; Turkey’s multiple comparison test). **, p < 0.01. (D-E) qRT-PCR (D; n=6 in each group; each point represents one technical replicate; two-way ANOVA; Turkey’s multiple comparison test; **, p < 0.01) and Western Blotting (E) were used to detect *Bcl2*, *Bax*, *Caspase-3*, and *Caspase-9* expression levels. Fifty oocytes for each group were collected for qRT-PCR or 100 oocytes for each group were collected for Western Blotting. (**F**) Cathepsin B levels of oocytes in Ctrl, NaF, FA, and MTX groups. Scale bar, 20μm. (**G**) Relative fluorescence intensity of Cathepsin B levels in Ctrl, NaF, FA, and MTX groups (n=11, 16, 13, 14; one-way ANOVA; Turkey’s multiple comparison test). *, p < 0.05; **, p < 0.01. (**H**) Representative images of apoptotic oocytes in Ctrl, NaF, FA, and MTX groups. Scale bar, 20μm. (**I**) Rate of apoptosis in Ctrl, NaF, FA, and MTX groups (n=3 in each group; each point is cumulative data from one independent experiment; Bootstrap method). *, p < 0.05; **, p < 0.01.

### FA supplementation restores spindle disruption and chromosome misalignment in NaF-treated mice

Because meiotic arrest is mainly caused by defective spindle structure, we examined this process in oocytes from the four groups. As assessed by immunostaining images, the rates of abnormal spindles and misaligned chromosomes were remarkably reduced in the NaF+FA group as compared with the NaF group (Fig. 3A-C), while treatment with MTX increased the meiotic defects in the NaF+FA group (Fig. 3A-C). In addition, transcriptional levels of several genes involved in meiotic progression including *Tuba1a* and *Nek2*, were significantly increased after FA treatment in NaF group (Fig. 3D-E). These results indicated that FA could restore the spindle disruption and chromosome aberrance of oocytes in NaF-treated mice.

### FA supplementation restores mitochondrial function in oocytes from NaF-treated mice

To verify the effect of FA administration on mitochondrial function in oocytes from NaF-treated mice, we assessed mitochondria distribution. As shown in Figure 4A, the rate of homogeneous distribution in NaF+FA significantly increased compared with that in the NaF group, whereas MTX reduced the rate of homogeneous distribution. The abnormal distribution of mitochondria indicates that oocyte function might be compromised by NaF administration. Given that the most important function of mitochondria is to produce ATP for cell development, ATP content was measured in oocytes from different groups. The results showed that ATP levels were recovered prominently in NaF+FA group compared with that in NaF group but declined following MTX supplementation (Fig. 4B). Furthermore, mitochondrial DNA (mtDNA) copy number per oocyte as well as GSH levels were measured in oocytes. It was found that both mtDNA copy number and GSH level were higher in NaF+FA group than in NaF group, which were decreased after MTX treatment (Fig. 4C-D). Next, we assessed mitochondrial membrane potential by JC-1 staining. Mitochondria with high membrane potential presented red fluorescence, while those with low membrane potential exhibited green fluorescence (Fig. 4E). Quantitative analysis revealed that the ratio of red to green signal was much stronger in NaF+FA group than that in NaF group but declined following MTX supplementation (Fig. 4F). These results suggested that FA repaired NaF-induced disruption to mitochondrial function to some extent.

### FA supplementation attenuates ROS levels to suppress apoptosis in oocytes from NaF-treated mice

Mitochondrial dysfunction is a known cause of ROS generation and oxidative stress; thus, we performed dichlorofluorescein (DCFH) staining to compare ROS levels among each group. The results showed that much weaker ROS signals appeared in NaF+FA group than in NaF group (Fig. 5A-B). By contrast, MTX supplementation effectively increased accumulated ROS in NaF+FA group (Fig. 5A-B). Several oxidative stress-related genes were detected using qRT-PCR and showed an opposite trend to that of ROS (Fig. 5C), which is consistent with previous studies [8, 29]. Since high levels of ROS usually speed up the process of apoptosis [30], we measured cell apoptosis in different groups. As expected, FA increased the Bcl2 levels inhibited by NaF treatment, which was suppressed by supplementation with MTX (Fig. 5D-E). In NaF-treated group, the remaining apoptosis-related genes were suppressed by FA both at mRNA (Fig. 5D) and protein levels (Fig. 5E, Supplementary Fig. 2); however, their expressions were restored by MTX supplementation. As a marker of poor-quality oocytes [31, 32], cathepsin B activity was measured in different groups. The results showed that cathepsin B activity in NaF+FA group decreased compared with that in NaF group and increased after MTX supplementation (Fig. 5F-G). The results were further confirmed by Annexin-V staining, FA led to a lower incidence of apoptosis in oocytes, which was recovered by supplementation with MTX (Fig. 5H-I). This finding was consistent with the above results that reduced mitochondria membrane potential, considered as a key indicator of apoptosis, could be rescued by FA.

We further used anti-FOLR antibodies to specifically block FA receptors, and the results showed that, after blocking FA receptors *in vivo*, the restoring effect of FA on oocytes from NaF-exposed mice were abolished, indicating that FA could indeed inhibit the effect of NaF (Supplementary Fig. 3).

### Administration of FA in vitro repairs meiotic defects and oocyte quality in NaF-exposed oocytes

**Figure 6.**
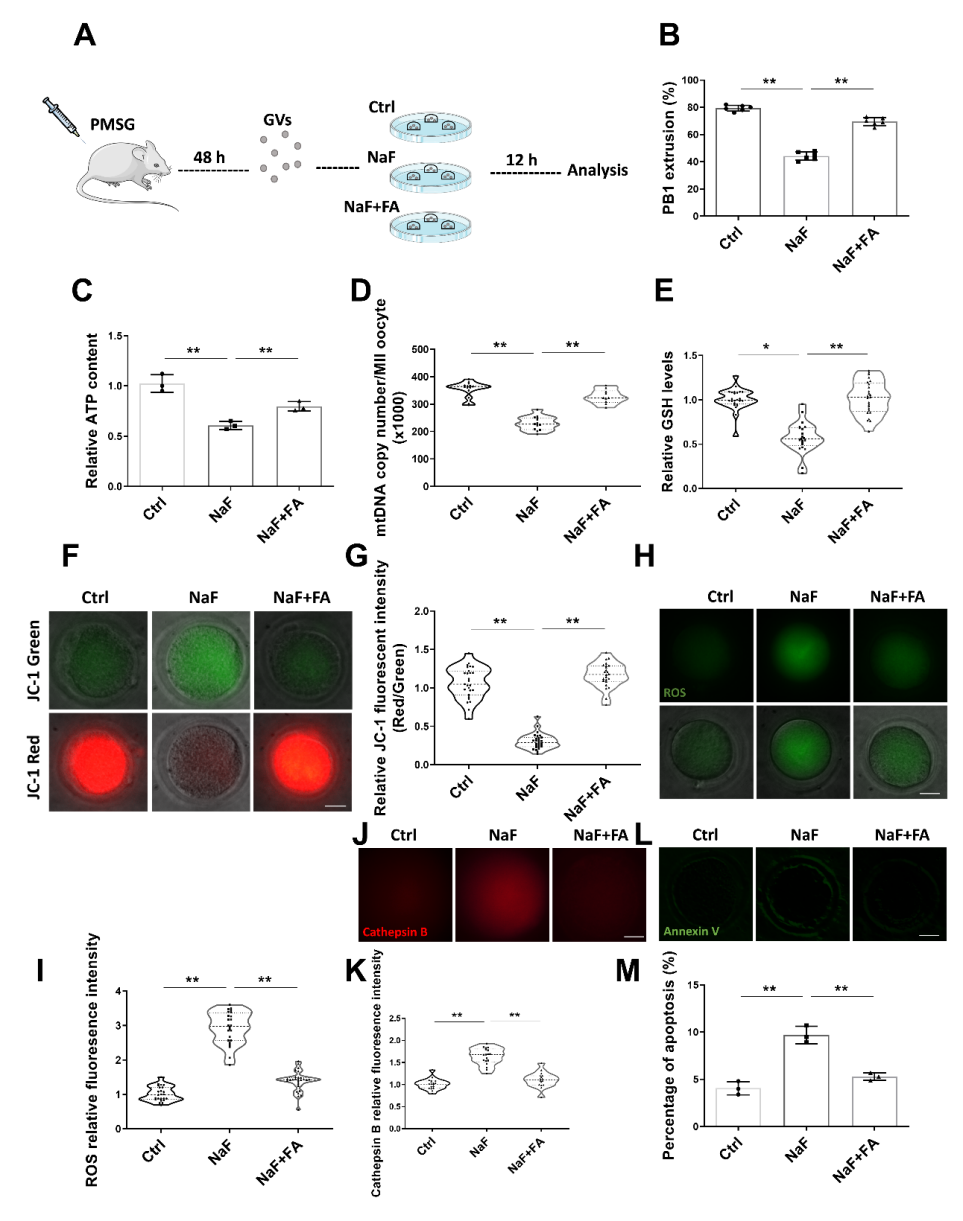
**Treatment of FA *in vitro* repairs meiotic defects and oocyte quality in NaF-exposed oocytes**. (**A**) Timeline diagram of NaF or NaF+FA supplementation to *in vitro* cultured oocytes. (**B**) PBE rates of oocytes in Ctrl, NaF, and NaF+FA groups (n=6 in each group; each point is cumulative data from one independent experiment; one-way ANOVA; Turkey’s multiple comparison test). **, p < 0.01. (**C**) Relative ATP content of oocytes exposed to Ctrl, NaF and NaF+FA groups (n=3 in each group; each point represents one independent experiment; Bootstrap method). **, p < 0.01. (**D**) MtDNA copy number of oocytes in Ctrl, NaF and NaF+FA groups (n=14, 13, 10; two-way ANOVA; Turkey’s multiple comparison test). **, p < 0.01. (**E**) Relative GSH level of oocytes in Ctrl, NaF, and NaF+FA groups (n=24, 21, 25; two-way ANOVA; Turkey’s multiple comparison test). *, p < 0.05; **, p < 0.01. (**F**) Mitochondrial membrane potential of oocytes. Scale bar, 20μm. (**G**) Relative fluorescence intensity of the mitochondrial membrane potential in Ctrl, NaF, and NaF+FA groups (n=27, 28, 24; one-way ANOVA; Turkey’s multiple comparison test). **, p < 0.01. (**H**) ROS levels of oocytes exposed to NaF and NaF+FA. Scale bar, 20μm. (**I**) Relative fluorescence intensity of ROS levels in Ctrl, NaF, and NaF+FA groups (n=24, 24, 28; one-way ANOVA; Turkey’s multiple comparison test). **, p < 0.01.(J) Representative images of Cathepsin B levels of oocytes exposed to NaF and NaF+FA. Scale bar, 20μm. (**K**) Relative fluorescence intensity of Cathepsin B levels in Ctrl, NaF, and NaF+FA groups (n=18, 18, 16; one-way ANOVA; Turkey’s multiple comparison test). **, p < 0.01. (**L**) Representative images of apoptosis of oocytes exposed to NaF and NaF+FA. Scale bar, 20μm. (**M**) Rate of apoptosis in Ctrl, NaF, and NaF+FA groups (n=3 in each group; each point is cumulative data from one independent experiment; Bootstrap method). **, p < 0.01.

Since *in vivo* administration of FA could ameliorate the quality of oocytes from NaF-treated mice by reducing the accumulation of ROS, we investigated whether supplementation of FA *in vitro* exerted similar effects. We treated oocytes in *in vitro* maturation medium and then assessed the development of oocytes (Fig. 6A). Consistent with the results from *in vivo* treatment, *in vitro* administration of NaF exhibited similar kinetics of meiotic progression by presenting a reduced proportion of PBE, which was restored following FA supplementation (Fig. 6B). Spindle/chromosome structures were also observed, and FA treatment rescued spindle assembly and chromosome alignment caused by NaF (Supplementary Fig. 4). Likewise, *in vitro* treatment of FA restored the abnormal mitochondrial functions in NaF-exposed oocytes, as evidenced by ATP content, mtDNA copy number, GSH level, and mitochondrial membrane potential (Fig. 6C-G). In addition, *in vitro* treatment of FA reduced ROS, Cathepsin B activity, and apoptosis of oocytes exposed to NaF (Fig. 6H-M). Altogether, these observations suggested that FA could suppress excessive ROS-induced apoptosis in NaF-exposed oocytes, thereby ameliorating the deterioration of oocytes.

### FA eliminates oocyte defects via the Sirt1/Sod2 pathway in NaF-treated mice

**Figure 7.**
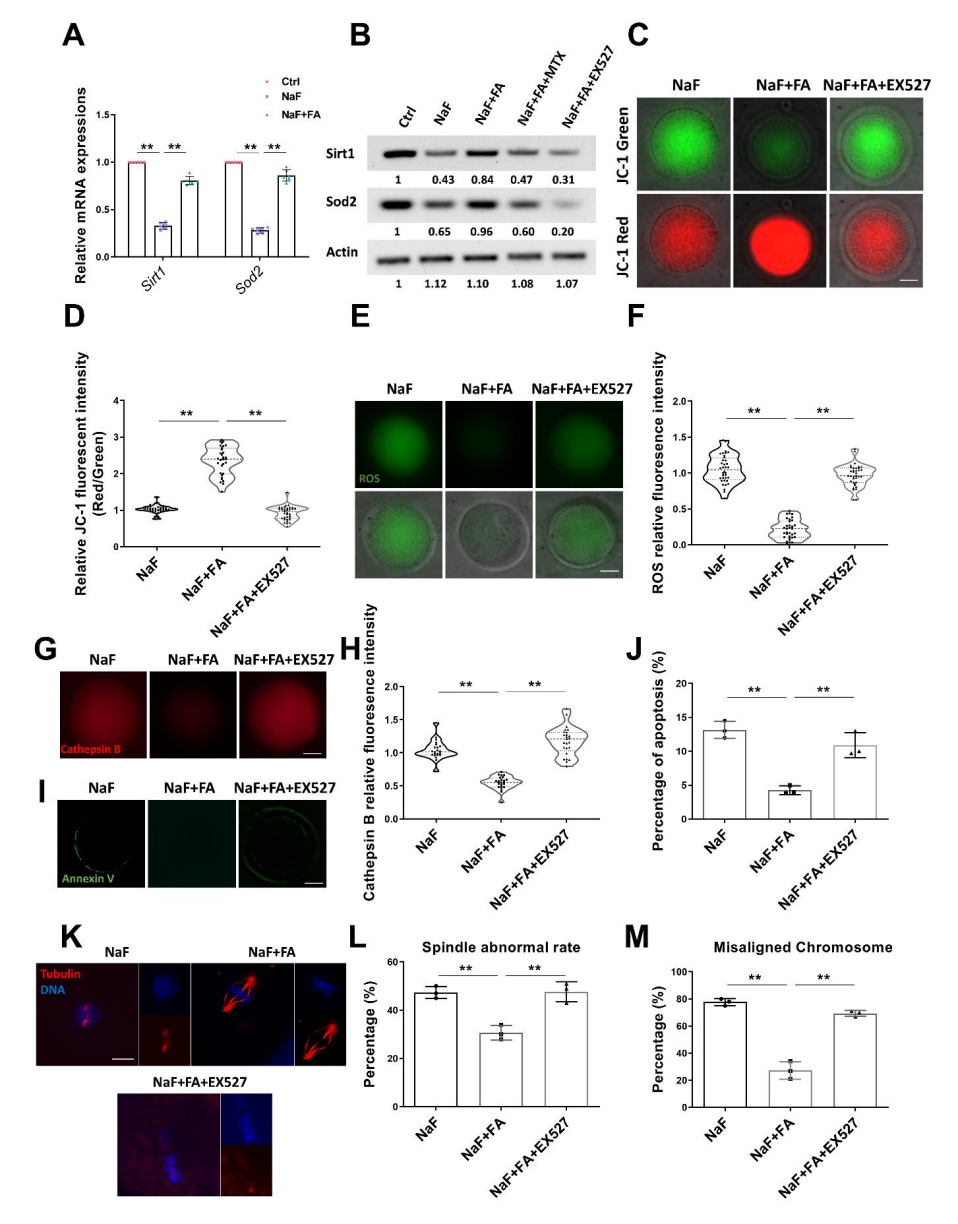
**FA eliminates oocyte defects via the *Sirt1*/*Sod2* pathway in NaF-treated mice**. (A-B) qRT-PCR (A; n=6 in each group; each point represents one technical replicate; two-way ANOVA; Turkey’s multiple comparison test; **, p < 0.01) and Western Blotting (B) were used to detect *Sirt1* and *Sod2* expression levels in oocytes exposed to NaF and NaF+FA. Fifty oocytes for each group were collected for qRT-PCR or 100 oocytes for each group were collected for Western Blotting. (**C**) Mitochondrial membrane potential of oocytes in NaF, FA, and EX527 groups. Oocytes in NaF+FA+EX527 group were cultured in M16 medium containing 20 μM EX527. Scale bar, 20μm. (**D**) Relative fluorescence intensity of the mitochondrial membrane potential in NaF, FA, and EX527 groups (n=33, 31, 34; one-way ANOVA; Turkey’s multiple comparison test). **, p < 0.01. (**E**) ROS levels of oocytes in NaF, FA, and EX527 groups. Scale bar, 20μm. (**F**) Relative fluorescence intensity of ROS levels in NaF, FA, and EX527 groups (n=36, 33, 37; one-way ANOVA; Turkey’s multiple comparison test). **, p < 0.01. (**G**) Representative images of Cathepsin B levels of oocytes exposed to NaF, FA, and EX527. Scale bar, 20μm. (**H**) Relative fluorescence intensity of Cathepsin B levels in NaF, FA, and EX527 groups (n=22, 24, 25; Bootstrap method). **, p < 0.01. (**I**) Representative images of apoptosis of oocytes in NaF, FA, and EX527 groups. Scale bar, 20μm. (**J**) Rate of apoptosis in NaF, FA, and EX527 groups (n=3 in each group; each point is cumulative data from one independent experiment; Bootstrap method). **, p < 0.01. (**K**) Representative images of spindle morphologies and alignment of chromosomes in NaF, FA, and EX527 groups. Scale bar, 10μm. Red, α-tubulin; blue, DNA. (**L**) Percentage of aberrant spindles in NaF, FA, and EX527 groups (n=3 in each group; each point is cumulative data from one independent experiment; Bootstrap method). **, p < 0.01. (**M**) Percentage of misaligned chromosomes in NaF, FA, and EX527 groups (n=3 in each group; each point is cumulative data from one independent experiment; Bootstrap method). **, p < 0.01.

Since *Sirt1* is a critical regulator of mitochondrial biogenesis, we asked whether *Sirt1* was involved in restoration of meiotic progression and quality in NaF-exposed oocytes by FA. We found that both the mRNA and protein levels of *Sirt1* were remarkably reduced in NaF-exposed oocytes compared with those of the control ones but elevated after FA supplementation (Fig. 7A-B, Supplementary Fig. 5A-B). Mitochondrial *Sod2* (Superoxide dismutase 2) is a major antioxidant enzyme scavenging cellular ROS [33], and is regulated by *Sirt1* [34]. To test whether the FA-mediated recovery of *Sirt1* expression affected the expression of *Sod2*, the mRNA and protein levels of *Sod2* were examined. The results showed that *Sod2* expression was reduced in NaF-exposed oocytes but restored in the NaF+FA-exposed ones (Fig. 7A-B), indicating that the expression trend of *Sod2* was highly consistent with the expression of *Sirt1*. Meanwhile, either inhibition of FA by MTX or inhibition of *Sirt1* by a specific inhibitor EX527 [35], did not change the expression level of *Sod2* in NaF-treated oocytes, suggesting that FA supplementation activates the *Sirt1*/*Sod2* pathway in NaF-treated oocytes.

We next tested whether *Sirt1* or *Sod2* inhibition affected mitochondrial membrane potential. Fluorescence staining and intensity quantification revealed that mitochondrial membrane potential was suppressed after EX527 treatment in NaF+FA group (Fig. 7C-D), and *Sod2* inhibition showed a similar result (Supplementary Fig. 5C-D). On the contrary, it was found that ROS levels and Cathepsin B activity were restored following EX527 treatment in NaF+FA group (Fig. 7E-H). Accordingly, the percentage of apoptosis was not rescued by FA when *Sirt1* was inhibited by EX527 (Fig. 7I-J).

We further evaluated spindle/chromosome structures in different groups and observed that *Sirt1* inhibition did not rescue the spindle assembly or chromosome alignment restored by FA supplementation (Fig. 7K-M). Collectively, these results indicated that FA might activate the *Sirt1*/*Sod2* pathway and eliminate excessive ROS accumulated in NaF-exposed oocytes, thus reducing the occurrence of apoptosis and improving oocyte quality.

## DISCUSSION

Excessive NaF intake has been shown to adversely affect reproduction both in females and males [8, 36]. As an essential B-complex vitamin, we proposed that FA could ameliorate the quality of NaF-exposed oocytes. To confirm the hypothesis and gain insights into the mechanisms underlying FA’s beneficial effects on oocyte quality in NaF-exposed mice, we used *in vitro* and *in vivo* treated oocytes as experimental models. We aimed to provide a more solid knowledge base for FA application targeting the restoration of oocytes quality during NaF exposure.

We first assessed the effect of NaF on the meiotic progression and fertilization ability of mouse oocytes. Previous studies have reported that meiotic defects commonly occur in NaF-exposed oocytes, which is a key indicator of oocyte quality [3, 7, 37]. Consistent with these observations, we found that NaF-exposed oocytes had a lower PBE rate, suggesting a harmful effect of NaF on oocytes. The supplementation of FA restored meiotic progression and fertilization ability, indicating that FA indeed has the potential to ameliorate oocyte quality. To further uncover how FA rescues the NaF-induced decline of oocyte development potential, we examined the critical indicators of oocyte quality. Meiotic spindle assembly is a critical cellular structure for accurate chromosomal distribution [38], and meiotic defect is mainly due to aberrant spindle/chromosome structure. Our data showed that FA supplementation recovered the spindle/ chromosome structure of NaF-exposed oocytes.

During oocyte maturation, MGCs and CCs play an important role in maintaining the appropriate proliferation, differentiation, and apoptosis of oocytes [39, 40]. NaF has been reported to exert an inhibitory effect on MGC apoptosis and CC expansion [7, 37, 41]. In this study, FA treatment significantly decreased NaF-induced MGC apoptosis, while neither NaF nor FA altered CC apoptosis. The explanation for this phenomenon may be that NaF treatment does not affect the apoptosis of CCs, or the apoptosis program in CCs has just initiated and cannot be detected yet.

As an important organelle that produces ATP in oocytes, mitochondrion is critical for oocyte development [42]. Mitochondrial dysfunction is highly related to oocyte fragmentation, fertilization failure, and mitochondria-driven apoptosis [43]. Thus, we tested mitochondrial distribution in NaF-exposed oocytes. Our findings confirmed that disturbed mitochondrial distribution may be an important reason for meiotic defects and arrest of oocyte development under NaF treatment. This supported the notion that NaF disrupts mitochondrial function in oocytes [3, 44], and subsequent analyses of ATP content, mtDNA copy number, GSH level, and mitochondrial potential demonstrated that FA could reverse the mitochondrial dysfunction induced by NaF in oocytes.

**Figure 8.**
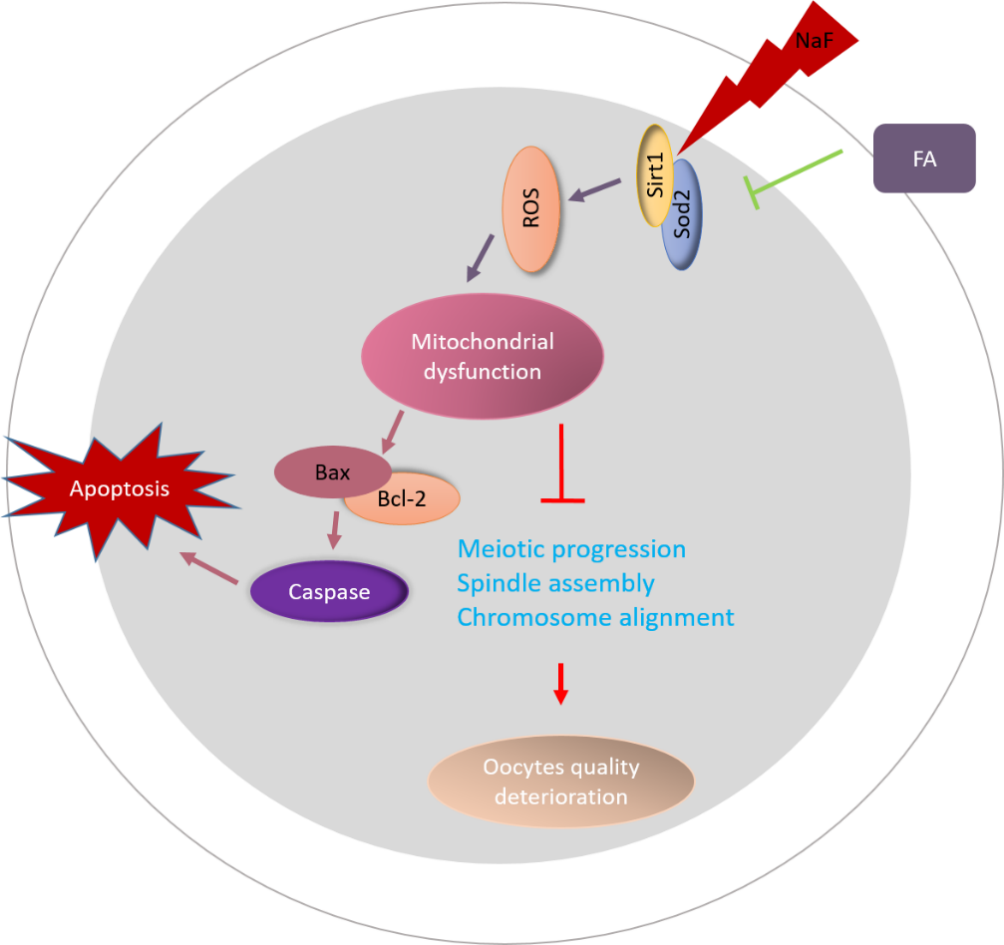
**Diagram illustrating the proposed mechanisms mediating the beneficial effects of FA on NaF-exposed oocytes**. FA supplementation induced *Sirt1* expression in oocytes from NaF treated mice, which in turn promoted the assembly of meiotic apparatus, ameliorated excessive ROS levels, and reduced apoptosis.

Compromised mitochondrial potential and function predict ROS accumulation and apoptosis in NaF-exposed oocytes, which accounts for oocyte fragmentation. Our results revealed that FA supplementation efficiently ameliorated excessive ROS and suppressed oocyte apoptosis. As a prominent lysosomal cysteine protease, cathepsin B could induce apoptosis and could be used as a marker of poor quality of oocytes and embryos [31, 32]. A previous study suggested that cathepsin B could promote the translocation of apoptosis-inducing components from mitochondria to cytosol, thus degrading mitochondrial membrane [45]. In the present study, an increased cathepsin B activity in NaF-exposed oocytes further highlighted the adverse impact of NaF on oocyte quality.

To test whether FA could recover the defects of NaF-exposed oocytes *in vitro*, germinal vesicle (GV) oocytes were co-incubated with NaF or NaF+FA and were used for analysis, and the results were consistent with those from the *in vivo* models. To ascertain the mechanisms of how FA rescues the quality of NaF-exposed oocytes, we tested the potential targets of FA in NaF-exposed oocytes. Recent reports have demonstrated the interplay between *Sirt1* and ROS via the *Sirt1*/*Sod2* pathway [46, 47]. Our results confirmed that in NaF-exposed oocytes, FA increased *Sirt1* expression, leading to ROS reduction and improving oocyte quality. NaF-induced suppression of *Sirt1* has also reportedly occurred in the testes of golden hamsters, F9 cells, and human neuroblastoma SH-SY5Y cells [8, 48, 49]. However, these observations were disrupted by EX527-mediated inhibition of *Sirt1* activity, and this indicated a critical role of *Sirt1* in mediating the amelioration of NaF-exposed oocyte quality by FA.

Collectively, we provided evidence from *in vivo* and *in vitro* experiments that FA supplementation improved the quality of NaF-exposed oocytes by promoting oocyte maturation and fertilization ability. In particular, FA restored mitochondrial function in NaF-exposed oocytes to reduce ROS accumulation and apoptosis. We further revealed that *Sirt1* recapitulated the benefits of FA supplementation. Overall, our study expounded a theoretical basis for application of FA to improve the fertility of females exposed to NaF environment.

## Supplementary Materials

The Supplementary data can be found online at: www.aginganddisease.org/EN/10.14336/AD.2022.0217.


