# Supplementary material for: Folic acid Ameliorates the Declining Quality of Sodium Fluoride-Exposed Mouse Oocytes through the Sirt1/Sod2 Pathway
Source: Aging Dis. 2022 Oct 1;13(5):1471–87. doi: 10.14336/AD.2022.0217 (PMC9466976; doi:10.14336/AD.2022.0217)
Supplement: Supplementary file 1 [file AD-13-5-1471-s.pdf]

SUPPLEMENTARY DATA

Folic acid Ameliorates the Declining Quality of Sodium Fluoride-Exposed Mouse Oocytes through the *Sirt1*/*Sod2* Pathway

Xiaoyuan Lin[1], Beibei Fu[1], Yan Xiong[1], Shiyao Xu[1], Jin Liu[2], Mohamed Y. Zaky[3], Dan Qiu[1, 4*], Haibo Wu[1*]

© 2022. Lin X et al. Published online at http://www.aginganddisease.org/EN/10.14336/AD.2022.0217

SUPPLEMENTARY DATA

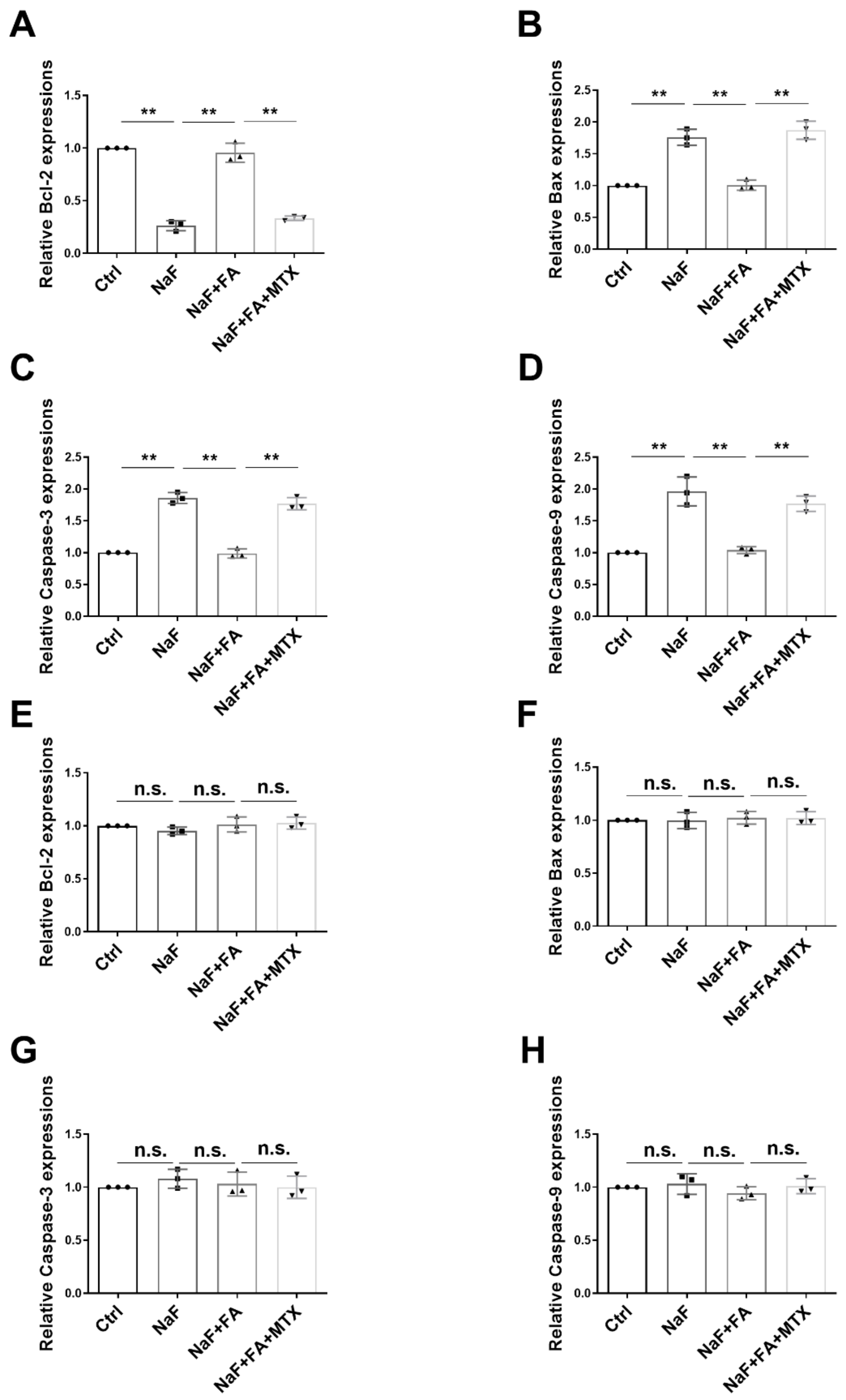

Supplementary Figure 1. Effects of FA on apoptosis of mural granulosa cells (MGCs) and cumulus cells (CCs) in NaF-treated mice. (A-I) Statistical analysis of Western Blotting in MGCs (**A**-**E**) and CCs (**F**-**I**). N=3 in each group; each point represents one independent experiment; Bootstrap method. **, $p < 0.01$; n.s., not significant.

© 2022. Lin X et al. Published online at http://www.aginganddisease.org/EN/10.14336/AD.2022.0217

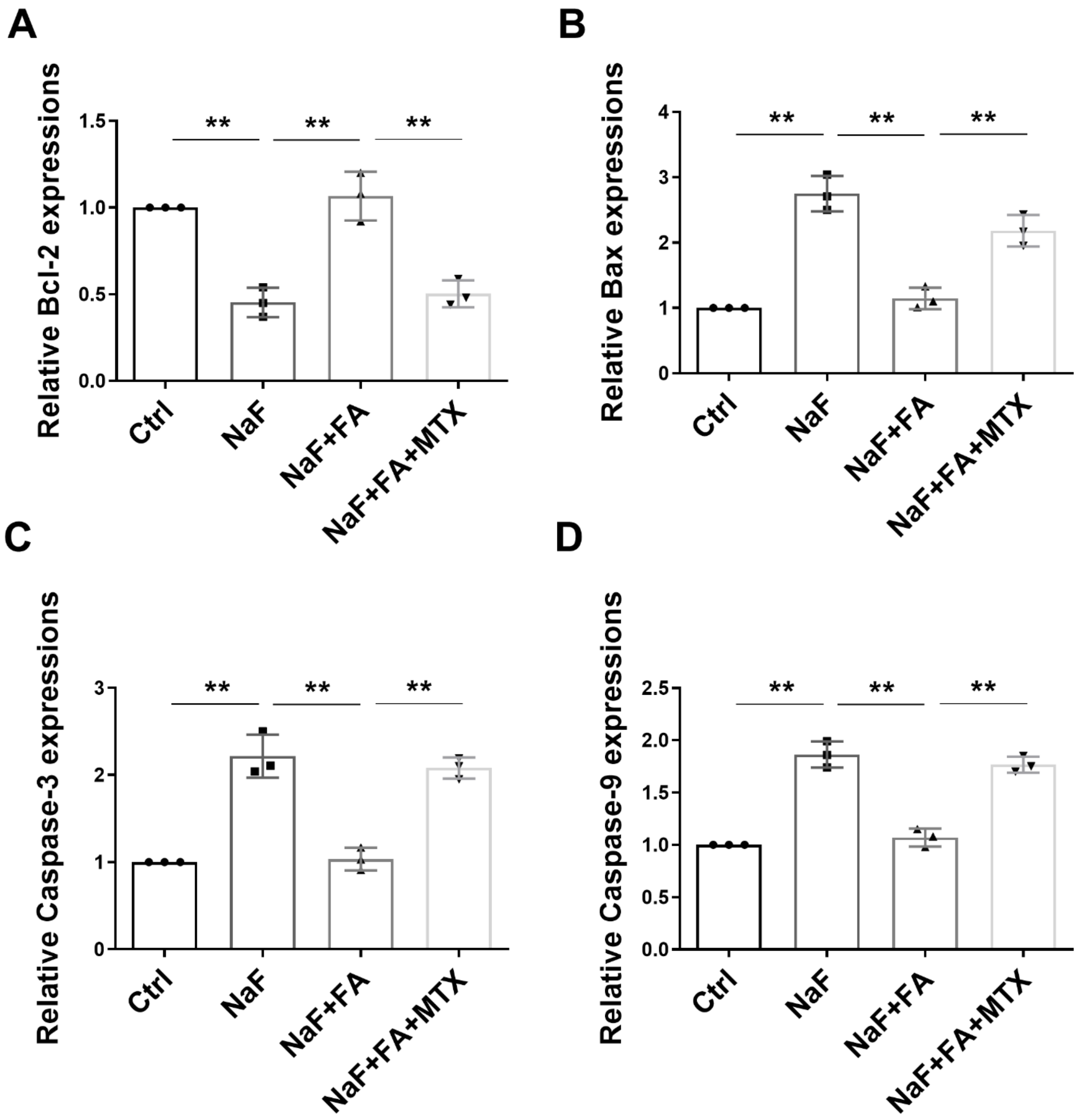

Supplementary Figure 2. FA attenuates ROS production to suppress cell apoptosis in NaF-treated mice. (A-D) Statistical analysis of Western Blotting in oocytes. N=3 in each group; each point represents one independent experiment; Bootstrap method. **, $p < 0.01$.

© 2022. Lin X et al. Published online at http://www.aginganddisease.org/EN/10.14336/AD.2022.0217

SUPPLEMENTARY DATA

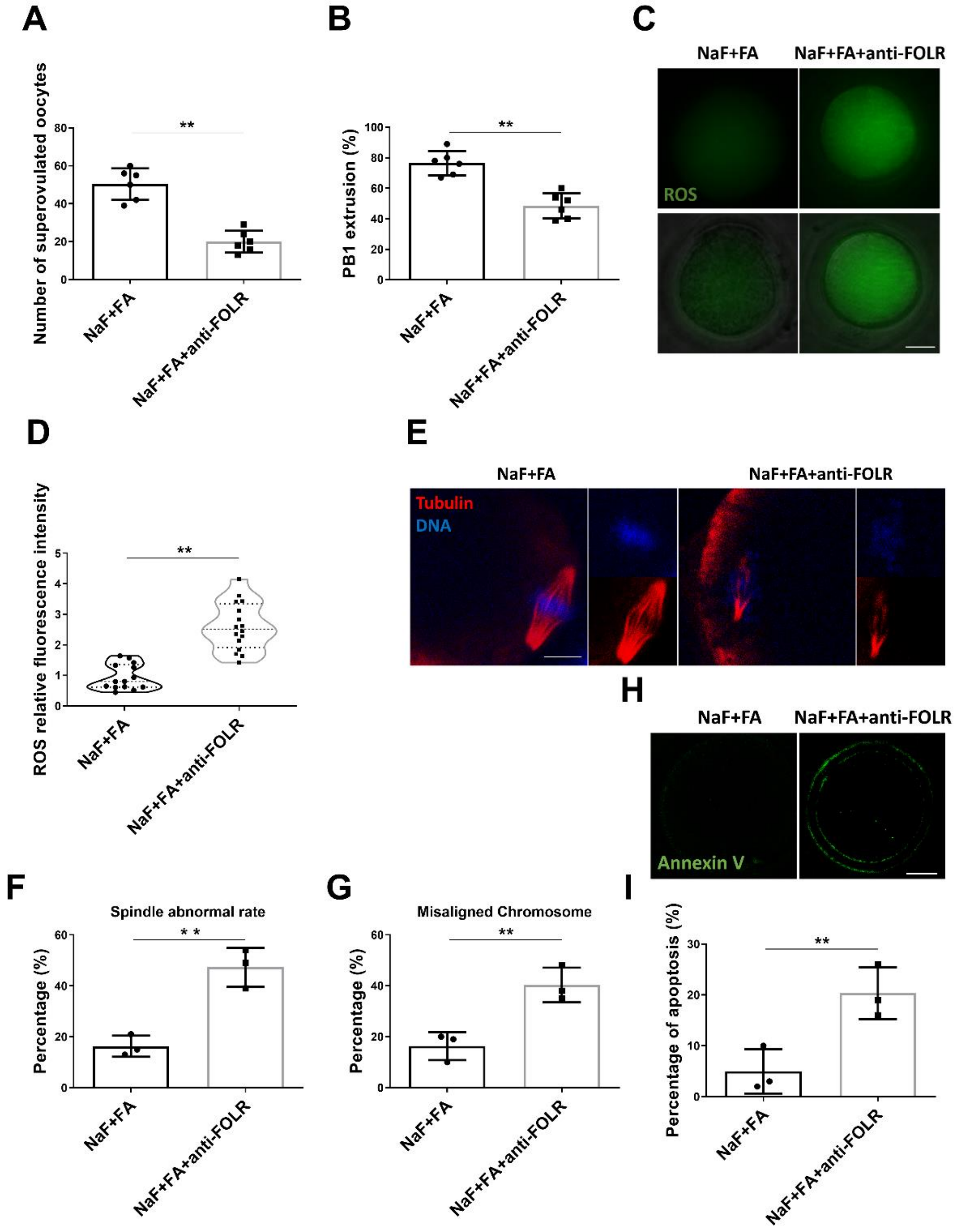

Supplementary Figure 3. FA blocking abolished the restoring effect of FA on oocytes from NaF-exposed mice. (A) Ovulated oocytes were counted in NaF+FA and NaF+FA+anti-FOLR mice (n=6 in each group; each point represents one mouse; two-tailed t test). **, $p < 0.01$. **(B)** PBE rates of oocytes from mice administered with NaF+FA or NaF+FA+anti-FOLR (n=6 in each group; each point is cumulative data from one independent experiment; two-tailed t test). **, $p < 0.01$. **(C)** ROS levels of oocytes in NaF+FA and NaF+FA+anti-FOLR groups. Scale bar, 20μm. **(D)** Relative fluorescence intensity of ROS levels in NaF+FA and NaF+FA+anti-FOLR groups (n=14, 16; two-tailed t test). **, $p < 0.01$. **(E)** Representative images of spindle morphologies and alignment of chromosomes in NaF+FA and NaF+FA+anti-FOLR groups. Scale bar, 10μm. Red, α-tubulin; blue, DNA. **(F)** Percentage of aberrant spindles in NaF+FA and NaF+FA+anti-FOLR groups (n=3 in each group; each point is cumulative data from one independent experiment; Bootstrap method). **, $p < 0.01$. **(G)** Percentage of misaligned chromosomes in NaF+FA and NaF+FA+anti-FOLR groups (n=3 in each group; each point is cumulative data from one independent experiment; Bootstrap method). **, $p < 0.01$. **(H)** Representative images of apoptotic oocytes in NaF+FA and NaF+FA+anti-FOLR groups. Scale bar, 20μm. **(I)** Rate of apoptosis in NaF+FA and NaF+FA+anti-FOLR groups (n=3 in each group; each point is cumulative data from one independent experiment; Bootstrap method). **, $p < 0.01$.

© 2022. Lin X et al. Published online at http://www.aginganddisease.org/EN/10.14336/AD.2022.0217

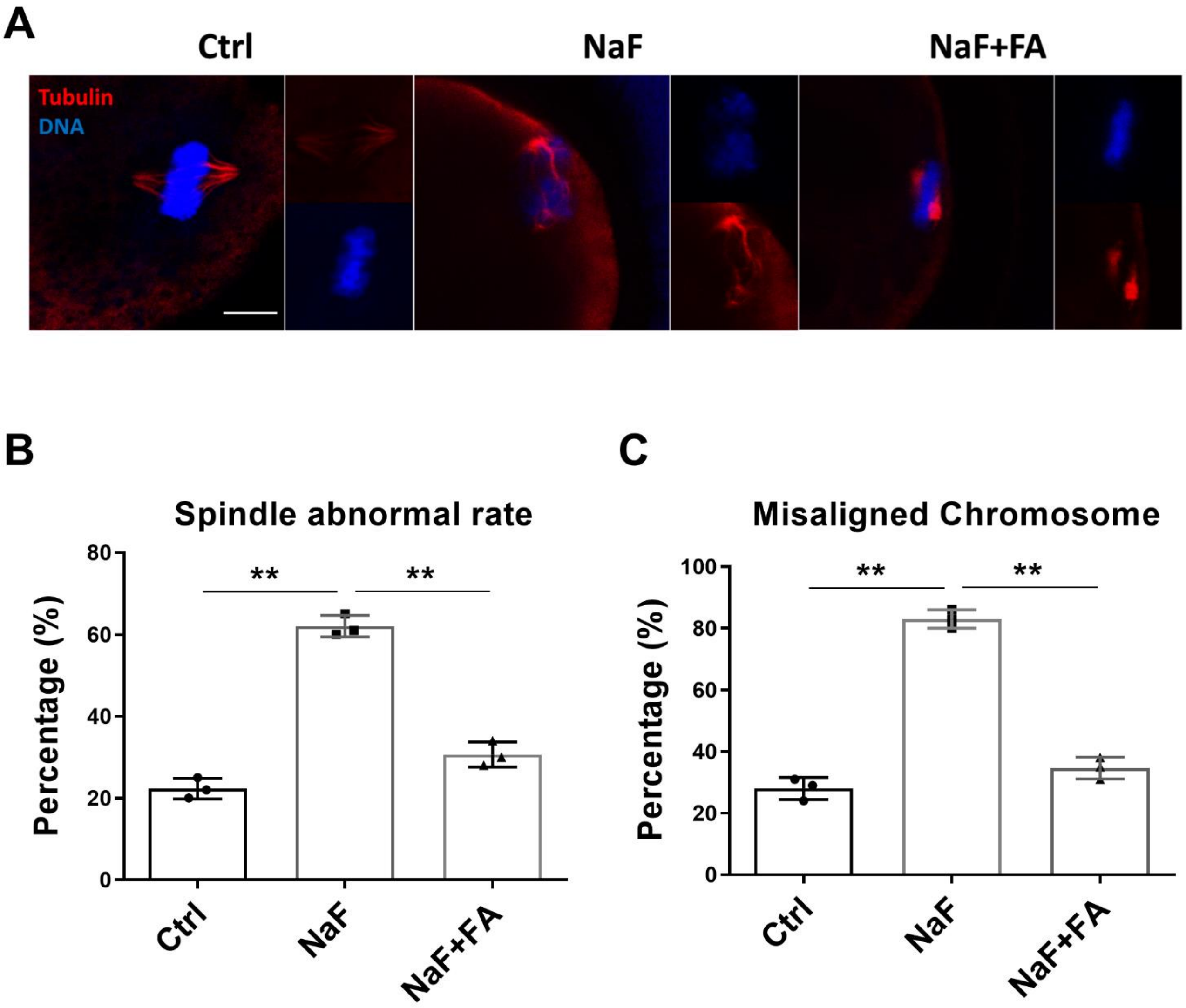

Supplementary Figure 4. Treatment of FA *in vitro* restores meiotic defects and oocyte quality in NaF-exposed oocytes. (A) Representative images of spindle morphologies and alignment of chromosomes in Ctrl, NaF, and FA groups. Scale bar, 10μm. Red, α-tubulin; blue, DNA. **(B-C)** Percentage of aberrant spindles in Ctrl, NaF, and NaF+FA groups (n=3 in each group; each point is cumulative data from one independent experiment; Bootstrap method). **, $p < 0.01$.

© 2022. Lin X et al. Published online at http://www.aginganddisease.org/EN/10.14336/AD.2022.0217

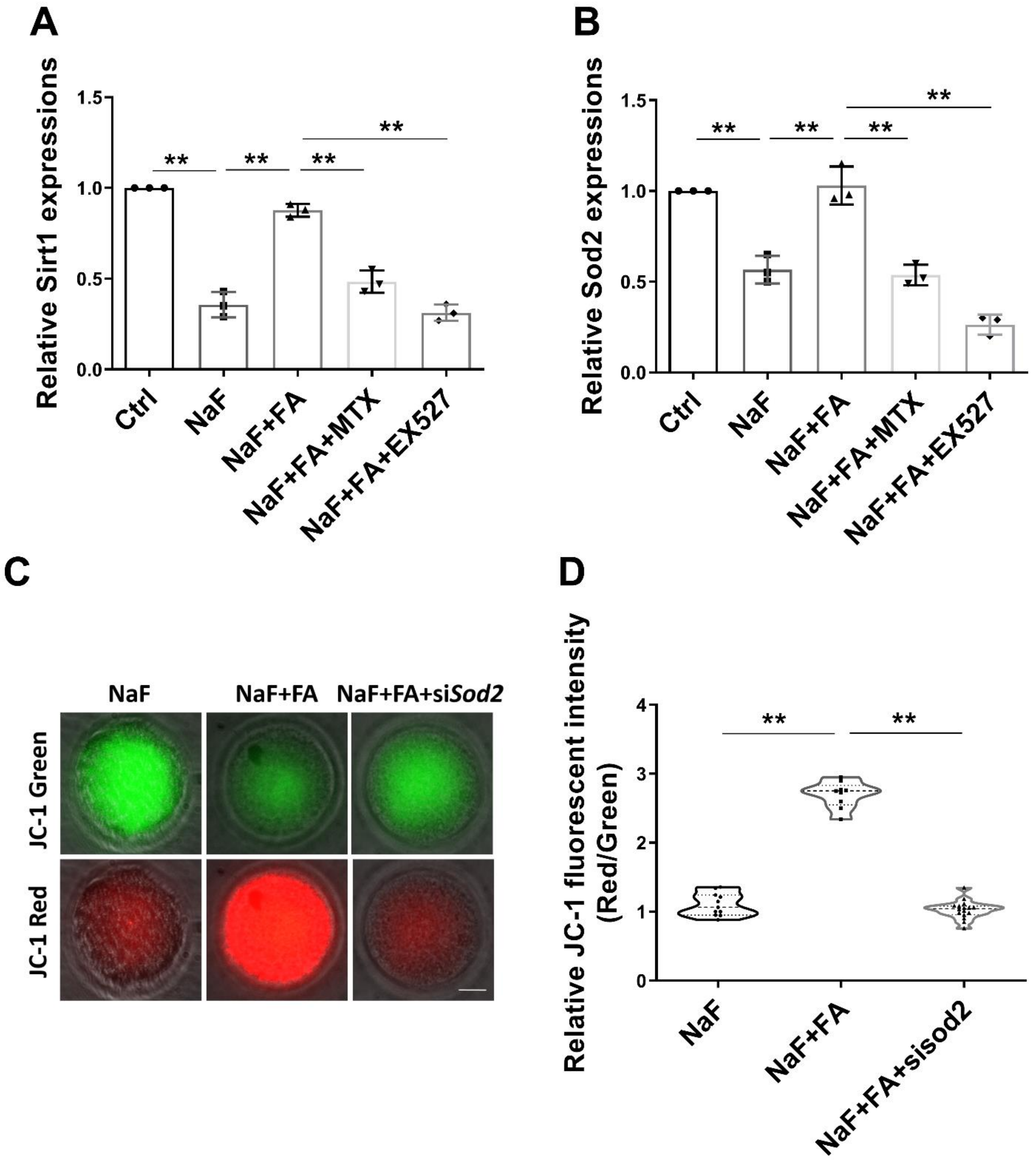

Supplementary Figure 5. eliminates oocytes defects via the *Sirt1*/*Sod2* pathway in NaF-treated mice. (A-B) Statistical analysis of Western Blotting in oocytes. N=3 in each group; each point represents one independent experiment; Bootstrap method. **, $p < 0.01$. **(C)** Mitochondrial membrane potential of oocytes in NaF, FA, and si*Sod2* groups. Scale bar, 20μm. **(D)** Relative fluorescence intensity of the mitochondrial membrane potential in NaF, FA, and si*Sod2* groups (n=11, 9, 16; one-way ANOVA; Turkey's multiple comparison test). **, $p < 0.01$.

© 2022. Lin X et al. Published online at http://www.aginganddisease.org/EN/10.14336/AD.2022.0217

SUPPLEMENTARY DATA

Supplementary Table 1. List of primers used for qRT-PCR.

Primers	Sequences (5’ – 3’)
Bcl2 F	TTCGGGATGGAGTAAACTGG
Bcl2 R	TGGATCCAAGGCTCTAGGTG
Bax F	ATGCGTCCACCAAGAAGCTGAG
Bax R	CCCCAGTTGAAGTTGCCATCAG
Caspase-3 F	GACTGGGATGAACCACGACCC
Caspase-3 R	TCTGACTGGAAAGCCGAAAC
Caspase-9 F	CTGGGAAGGTGGAGTAGGAC
Caspase-9 R	GCGGTGGTGAGCAG
Tuba1a F	TCGTATCCACTTCCCTCTGG
Tuba1a R	ACTGGATGGTACGCTTGGTC
Nek2 F	ATACTGTGAGGGAGGGGACC
Nek2 R	AGTCCCCCAGCTTGACATTG
Sod1 F	GGGTTCCACGTCCATCAGTA
Sod1 R	TTGCCCAGGTCTCCAACAT
Sod2 F	CAGACCTGCCTTACGACTATGG
Sod2 R	CTCGGTGGCGTTGAGATTGTT
Cat F	CAGCGACCAGATGAAGCAGT
Cat R	CCTCAAAGTATCCAAAAGCACC
Gpx1 F	CCGCTTTCGTACCATCGACA
Gpx1 R	CGCCCATCTGAGGGGATTTT
Prdx6 F	CGCCAGAGTTTGCCAAGAG
Prdx6 R	TCCGTGGGTGTTTCACCATTG
Sirt1 F	TGCTGCAGACGTGGTAATGT
Sirt1 R	TGGTGAAGATCTATGCAGGCTC
β-actin	TCGTGGGCCGCCCTAGGCAC
β-actin	TGGCCTTAGGGTTCAGGGGGG

© 2022. Lin X et al. Published online at http://www.aginganddisease.org/EN/10.14336/AD.2022.0217
